# Exploring the interplay of personality traits, academic burnout, and academic engagement in dental students

**DOI:** 10.1186/s12909-024-06479-8

**Published:** 2025-04-23

**Authors:** Jaehee Rho, Gieun Nam, Yongmin Shin, Yeeun Byeon, Jun-Young Lee, Yun-Jeong Kim, Jungjoon Ihm

**Affiliations:** 1 Department of Education, College of Educational Sciences, Yonsei University, Seoul, Korea; 2 Department of Psychiatry, SMG-SNU Boramae Medical Center, Seoul, Korea; 3 Department of Psychology, Gachon University, Seoul, Korea; 4 Department of Medical Device Development, College of Medicine, Seoul National University, Seoul, Korea; 5 Department of Periodontology, Seoul National University Gwanak Dental Hospital, Seoul, Korea; 6 Department of Dental Education, Seoul National University, Seoul, Korea; 7 Dental Research Institute, School of Dentistry, Seoul National University, 1, Gwanak-ro, Gwanak-gu, Seoul, 08826 Korea

**Keywords:** Personality traits, Academic burnout, Academic engagement, Emotion regulation, Dental students

## Abstract

**Background:**

This study aimed to investigate the relationship between personality traits, academic burnout, and academic engagement among dental students with emotion regulation as a mediating role. It sought to identify personality predictors within the HEXACO model and compare how these traits directly and indirectly affect students’ academic engagement and burnout.

**Methods:**

The participants were 228 dental students from School of Dentistry. Data were collected using the HEXACO personality inventory, the Emotion Regulation Questionnaire (ERQ), the Maslach Burnout Inventory-Student Survey (MBI-SS), and the Korean Academic Engagement Inventory (KAEI). The study employed structural equation modeling to explore the direct and mediated relationships between personality traits, emotion regulation, academic burnout, and academic engagement.

**Results:**

The findings indicated that specific personality traits, notably Extraversion, directly and indirectly influence both academic burnout and engagement, with emotion regulation serving as a mediating factor. Extraversion affected engagement directly and indirectly through cognitive reappraisal. Moreover, the burnout model revealed that, besides Extraversion, Agreeableness and Openness to experience also had a direct impact on burnout, suggesting a broader range of personality traits influencing burnout compared to engagement.

**Conclusions:**

The study underscores the significant role of personality traits, particularly Extraversion, in determining academic burnout and engagement, with emotion regulation often playing a mediating role. The findings of this study indicate that personality traits can be approached in a distinct manner when developing strategies to mitigate burnout and increase engagement among dental students. These insights could inform targeted interventions aimed at improving academic engagement and performance.

## Background

Healthcare professionals may experience stress, which can lead to burnout [1]. Burnout can negatively impact dentists’ clinical practice effectiveness, their performance in relation to patient safety, and their own health and well-being. Some studies have reported that healthcare students may experience a form of academic burnout due to the demanding educational curriculum and heavy practice load they face as students, which may potentially affect their professional careers adversely [1–3]. To cultivate healthy professionals, it is important to address the issue of academic burnout from the student stage. Exploring personality and emotion regulation factors associated with academic burnout can help reduce it and improve academic engagement.

Over the past decade, student burnout has frequently been cited as a factor contributing to maladaptive behaviors, which are defined as ineffective responses to stress that can hinder academic and social functioning [4]. Academic adjustment, on the other hand, involves effectively regulating study behaviors, maintaining intrinsic motivation to learn, and experiencing satisfaction with one’s degree program and academic performance [5]. Student burnout constitutes a psychological syndrome resulting from prolonged exposure to school-related stressful events and pressure to achieve [6]. It is known to be associated with delayed education, lower educational aspirations, academic achievement, occupation [4, 5], school engagement [6], and depressive symptoms [7]. Students experiencing school burnout often exhibit irresponsible behaviors such as a lack of interest in class activities and teachers, repeated absences, and tardiness, all of which negatively impact the overall classroom atmosphere [6]. Additionally, students experiencing burnout are significantly more likely to face burnout in the workplace [8]. This underscores the importance of implementing appropriate interventions, as they can enhance academic performance, prevent depressive symptoms, and reduce professional burnout, ultimately contributing to the development of a more resilient workforce [8].

Academic burnout is a state of physical and mental exhaustion resulting from academic-related stress, characterized by feelings of hostility and alienation. In contrast, academic engagement is defined by a high level of energy and mental resilience for learning [6, 9]. Given these characteristics, academic engagement and burnout are often considered opposing concepts. However, there is ongoing debate in the literature, with some researchers suggesting that burnout and engagement may not be strictly opposites but rather distinct constructs with complex interrelationships [10]. Previous research has established academic engagement as a significant predictor of positive outcomes in healthcare profession education, notably in academic achievement [11]. It is understood as a stable, affective-cognitive state extending beyond specific tasks, encompassing vigor, dedication, and absorption. Given that high levels of burnout and low levels of engagement predict poor long-term educational outcomes [12], it is crucial to identify student outcomes that signal an increased risk of maladjustment early and to alert educators accordingly.

Personality stands out among the variables that predict academic burnout and engagement [12]. It is generally considered the most fundamental individual difference factor in understanding and predicting behavior across various contexts, often interacting with the environment to influence outcome variables [13, 14]. While dental education researchers continue to explore psychosocial factors affecting academic achievement in dental students [15–17], there remains a significant oversight in healthcare education, particularly in the dental and medical fields, regarding how students’ personal traits influence their learning [12].

Given that personality is the foundational factor distinguishing individuals and impacting nearly all outcome variables, considering personality-adaptive educational treatments becomes crucial. Previous meta-analyses have highlighted personality’s significant impact on academic performance, emphasizing that it is not merely an adjunct to intelligence [18, 19]. In a longitudinal study of medical students, it was found that high-risk students for psychological health deterioration exhibited high conscientiousness [18]. Conversely, students who were more resilient to stress tended to display high extroversion and low conscientiousness [18]. Additionally, Extraversion personality among medical students exhibited a strong correlation with academic engagement and academic burnout, with high Extraversion associated with low burnout and heightened academic engagement [18].

In previous studies, personality has been assessed using the Five Factor Personality Model and the HEXACO model, proposed by Ashton and Lee [20], as a response to the limitations of the former. The HEXACO model incorporates previously unrecognized personality dimensions, comprising six factors: Honesty-Humility (e.g. sincerity, fairness, greed-avoidance, modesty), Emotionality (e.g. fearfulness, anxiety, dependence, sentimentality), Extraversion (e.g. expressiveness, social boldness, sociability, liveliness), Agreeableness (e.g. forgiveness, gentleness, flexibility, patience), Conscientiousness (e.g. organization, diligence, perfectionism, prudence), and Openness to experience (e.g. aesthetic appreciation, inquisitiveness, creativity, unconventionality) [21]. Research suggests that the HEXACO model offers practical and theoretical advantages over the Big Five in predicting individually, organizationally, and academically relevant outcomes [21, 22]. For example, Honesty-Humility captures personality facets such as sincerity and modesty, which are inadequately represented in Big Five scales [22]. Within academia, studies have demonstrated that HEXACO contribute to incremental variance in student outcomes such as GPA and conduct violations, surpassing the predictive power of the Big Five [23].

Emotion regulation involves modulating emotions to achieve desirable states or outcomes, with the main approach highlighted by Gross [24] being a process-oriented approach. This conceptual analysis examines the processes underlying various emotion regulation behaviors, explaining them in terms of information processing, including temporal scope. The first phase of emotion regulation is antecedent-focused regulation, primarily through cognitive reappraisal, while the second phase is response-focused regulation, mainly involving suppression. Cognitive reappraisal entails altering one’s interpretation of a situation to change its emotional impact, whereas suppression involves masking outward expressions, such as smiling despite stress [25]. Previous studies suggested emotion regulation also can influence students’ academic burnout and academic outcomes [26], doctor’s emotional exhaustion [27], burnout [1, 28, 29], and psychological well-being [28, 29].

The theory of emotional intelligence suggests that individuals with greater emotion regulation skills have a wider range of strategies for maintaining appropriate emotions, which helps them reduce or adapt to undesirable emotions in themselves and others [29]. A study on a non-physician population reported that individuals with low emotion regulation abilities may be more vulnerable to occupational burnout [30]. Research in the nursing profession has also shown that emotional regulation are negatively correlated with occupational stress levels and burnout [31, 32]. Yet, the role of emotion regulation in academic engagement and burnout among pre-health professions remains unclear.

The objective of this study is to explore the correlation between dental students’ personality traits and their academic engagement and burnout. Specifically, the research aims to identify which personality traits significantly predict academic engagement and burnout among these students. Additionally, the study will utilize structural equation modeling to uncover the mechanisms mediating emotion regulation in this relationship. A comparative analysis of the hypothesized models concerning academic engagement and burnout will be conducted to delineate commonalities and disparities. Such a comparison will provide valuable insights into the relationship between academic engagement and burnout, aiding in the design of targeted interventions to prevent academic burnout and promote engagement.

## Methods

### Participants

The study, which conducted surveys from April to July 2021, received approval from the Institutional Review Board (IRB No. S-D20210016) of the Ethics Committee at the School of Dentistry, Seoul National University. Participants were recruited from School of Dentistry and provided informed consent before voluntarily participating. Questionnaires were distributed to 320 students in total, with 228 consenting and participating, resulting in a participation rate of 71.25%. Of the 228 dental students who completed the questionnaires, 118 (51.8%) were male and 110 (48.2%) were female, with an average age of 22.43 years (range: 18–36, SD = 3.12). Of the participants, 82 (36.0%) were in the undergraduate program, and 146 (64.0%) were in the professional master’s program.

### Measures

We employed a comprehensive approach to assess various aspects of dental students’ personalities, emotion regulation, academic engagement and academic burnout through the administration of multiple self-report questionnaires. Personality traits were evaluated using the HEXACO Personality Inventory, comprising a 60-item version capable of assessing six distinct personality dimensions [33]. In our study, we utilized the Korean version of the inventory, validated by Yoo, Lee, and Ashton [34]. The HEXACO model incorporates previously unrecognized personality dimensions, comprising six factors: Honesty-Humility (HOHUM), Emotionality (EMOTI), Extraversion (EXTRA), Agreeableness (AGREE), Conscientiousness (CONSC), and Openness to experience (OPENN). Each domain of personality encompassed 10 items, and participants provided responses on a 5-point Likert scale, ranging from 1 (strongly disagree) to 5 (strongly agree), enabling a detailed examination of their personality characteristics.

To gauge emotion regulation among participants, we employed the Emotion Regulation Questionnaires (ERQ) developed by Gross and John [35]. This tool comprises 10 items and encompasses two distinct subscales: cognitive reappraisal (ER_reap), comprising six items, and suppression (ER_supp), consisting of four items. For example, cognitive reappraisal is illustrated by the statement: “When I want to feel less negative emotion, I change the way I’m thinking about the situation.” In contrast, suppression is represented by: “When I am feeling negative emotions, I make sure not to express them.” Participants rated each item on a 7-point Likert scale, providing nuanced insights into their emotion regulation strategies. The Korean adaptation of the ERQ, translated and validated by Shon [36], was utilized in our study, ensuring cultural and linguistic relevance for our sample population.

Academic engagement was evaluated using the Korean Academic Engagement Inventory (KAEI), consisting of 16 items divided into four subscales: dedication, vigor, efficacy, and absorption. The KAEI, developed and validated by Lee and Lee [37], is based on the engagement concept proposed by Schaufeli et al. [10]. Participants rated their level of academic engagement on a five-point Likert-type scale, ranging from 1 (strongly disagree) to 5 (strongly agree). The inventory covers various aspects of academic engagement, including dedication (e.g., “I feel proud when I study”), vigor (e.g., “I get energy when I study”), efficacy (e.g., “I have confidence in my studies”), and absorption (e.g., “Time flies when I study”).

To assess academic burnout among participants, we utilized the Maslach Burnout Inventory-Student Survey (MBI-SS), originally developed by Schaufeli et al. [10]. This instrument has been adapted and validated for use in the Korean context by Shin et al. [38] We purchased the MBI license to administer the instrument via the MindGarden webpage (http://www.mindgarden.com). The MBI-SS comprises 15 items organized into three dimensions: emotional exhaustion, cynicism, and academic efficacy. Participants rated their agreement with each item on a Likert scale ranging from 1 (strongly disagree) to 5 (strongly agree), facilitating nuanced assessments of their burnout levels across various dimensions.

### Data analysis

In this study, we employed item parceling, a technique that involves aggregating items and utilizing these aggregates as indicators of latent constructs, for structural equation modeling [39]. Initially, we conducted confirmatory factor analysis (CFA) using Mplus, following the procedures outlined by Brown [40], to confirm the designated factor structure of each construct, including academic burnout and engagement. Both CFA and structural equation modeling (SEM) analyses were conducted using maximum likelihood with robust standard errors (MLR) estimates. We assessed the fit of the hypothesized models using approximate fit indices, including the root mean square error of approximation (RMSEA), comparative fit index (CFI), and standardized root mean square residual (SRMR). The criteria for good fit were defined as RMSEA < 0.06, CFI > 0.95, and SRMR < 0.08 [41–43].

## Results

### Confirmatory factor analysis (CFA)

To ensure the integrity and reliability of the dataset, we removed eight responses identified as unfaithful or outliers. Based on the factor structure, CFA was conducted using 220 samples. The fit indices fell within acceptable ranges. The factor loadings obtained from the CFA are listed in Table 1. The factor rho reliabilities [44] were calculated as 0.762 for Honesty-Humility, 0.696 for Emotionality, 0.642 for Extraversion, 0.796 for Agreeableness, 0.843 for Conscientiousness, and 0.723 for Openness, indicating consistent measurement of the six factors. Factor loadings exceeding 0.45 confirmed convergent validity for each factor [40]. Additionally, discriminant validity was established by obtaining factor correlations lower than 0.80, as shown in Table 2.

**Table 1:** Structural equation model estimates of academic engagement and burnout

Variable	Academic engagement				Academic burnout			
	Estimates (b)	SE	Std Estimates	*p*-value	Estimates (b)	SE	Std Estimates Estimate	*p*-value
**ER_reap on**								
**HOHUM**	0.082	0.130	0.056	0.528	0.081	0.132	0.056	0.536
**EMOTI**	0.242	0.147	0.182	0.139	0.262	0.147	0.202	0.097
**EXTRA**	0.687	0.192	0.459	0.000	0.740	0.206	0.473	0.000
**AGREE**	0.451	0.175	0.233	0.006	0.452	0.178	0.232	0.007
**CONSC**	−0.046	0.116	−0.032	0.690	−0.058	0.118	−0.040	0.623
**OPENN**	0.019	0.064	0.020	0.771	0.006	0.090	0.005	0.943
**ER_supp on**								
**HOHUM**	−0.176	0.161	−0.108	0.266	−0.162	0.160	−0.099	0.306
**EMOTI**	−0.081	0.170	−0.055	0.624	−0.095	0.168	−0.066	0.561
**EXTRA**	−0.452	0.212	−0.270	0.025	−0.505	0.230	−0.289	0.020
**AGREE**	0.837	0.242	0.387	0.000	0.859	0.244	0.396	0.000
**CONSC**	0.081	0.140	0.050	0.561	0.073	0.141	0.045	0.604
**OPENN**	−0.041	0.100	−0.039	0.668	−0.170	0.108	−0.107	0.109
**Engagement on**								
**HOHUM**	−0.028	0.085	−0.030	0.744	-0.049	0.040	−0.107	0.217
**EMOTI**	0.195	0.101	0.228	0.087	0.026	0.044	0.063	0.562
**EXTRA**	0.605	0.159	0.629	0.000	−0.274	0.076	−0.560	0.000
**AGREE**	−0.072	0.120	−0.058	0.546	0.116	0.061	0.191	0.046
**CONSC**	0.060	0.075	0.065	0.423	−0.049	0.036	−0.108	0.174
**OPENN**	0.081	0.050	0.137	0.053	−0.065	0.028	−0.147	0.015
**Engagement/Burnout on**								
**ER_reap**	0.106	0.054	0.165	0.050	−0.015	0.025	−0.047	0.552
**ER_supp**	0.026	0.039	0.045	0.509	0.014	0.019	0.050	0.461

Note. HOHUM: Honesty-Humility, EMOTI: Emotionality, EXTRA: Extraversion, AGREE: Agreeableness, CONSC: Conscientiousness, OPENN: Openness to experience, ER_reap: cognitive reappraisal of Emotion Regulation, ER_supp: suppression of Emotion Regulation

**Table 2:** Factor correlation matrix for the latent variables

Factor	HOHUM	EMOTI	EXTRA	AGREE	CONSC
EMOTI	0.164				
EXTRA	0.121	−0.513			
AGREE	0.177	−0.238	0.455		
CONSC	0.416	−0.071	0.145	−0.042	
OPENN	0.133	−0.056	0.118	0.182	−0.035

Note. HOHUM: Honesty-Humility, EMOTI: Emotionality, EXTRA: Extraversion, AGREE: Agreeableness, CONSC: Conscientiousness, OPENN: Openness to experience

### Structural equation analysis of academic engagement

**Fig. 1:**
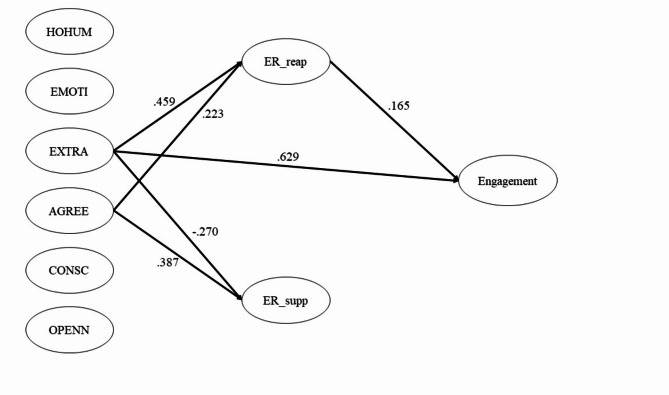
Structural equation model of academic engagement *Note.* HOHUM: Honesty-Humility, EMOTI: Emotionality, EXTRA: Extraversion, AGREE: Agreeableness, CONSC: Conscientiousness, OPENN: Openness to experience, ER_reap: cognitive reappraisal of Emotion Regulation, ER_supp: suppression of Emotion Regulation

The hypothesized model was fitted, and its fit indices fell within the good range: CFI = 0.900, SRMR = 0.063, and RMSEA = 0.062. In Table 1; Fig. 1, Extraversion was significantly positively associated with ER_reap (*b* = 0.687, *p* < .001), while Agreeableness was also significantly positively associated with ER_reap (*b* = 0.451, *p* = .006). However, the other personality variables did not affect ER_reap. Additionally, both Extraversion (*b* = − 0.452, *p* = .025) and Agreeableness (*b* = 0.837, *p* < .001) were significantly associated with ER_supp. Nevertheless, the other personality variables did not influence ER_supp. Furthermore, Extraversion (*b* = 0.605, *p* < .001) was found to be significantly positively associated with academic engagement, whereas ER_reap demonstrated a significantly positive association with academic engagement (*b* = 0.106, *p* = .050), while ER_supp did not exhibit a significant association with academic engagement (*b* = 0.026, *p* = .509). The only significant effect was the indirect path from Extraversion to academic engagement, mediated by ER_reap (*b* = 0.073, *p* < .05).

### Structural equation analysis of academic burnout

**Fig. 2:**
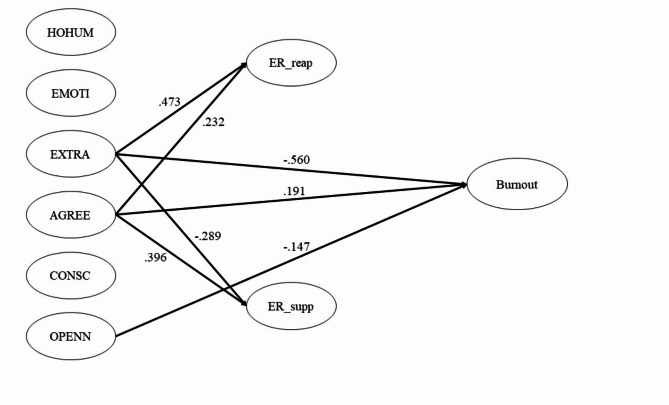
Structural equation model of academic burnout *Note.* HOHUM: Honesty-Humility, EMOTI: Emotionality, EXTRA: Extraversion, AGREE: Agreeableness, CONSC: Conscientiousness, OPENN: Openness to experience, ER_reap: cognitive reappraisal of Emotion Regulation, ER_supp: suppression of Emotion Regulation

In Table 1; Fig. 2, the hypothesized model was fitted, and its fit indices were within the good range: CFI = 0.908, SRMR = 0.064, and RMSEA = 0.059. Extraversion was significantly positively associated with ER_reap (*b* = 0.740, *p* < .001), while Agreeableness also showed a significantly positive association with ER_reap (*b* = 0.452, *p* = .007). However, the other personality variables did not affect ER_reap. Furthermore, both Extraversion (*b* = − 0.505, *p* = .020) and Agreeableness (*b* = 0.859, *p* < .001) were significantly associated with ER_supp. Additionally, Extraversion (*b* = − 0.274, *p* < .001) and Openness (*b* = − 0.065, *p* < .05) were found to be significantly negatively associated with academic burnout. Agreeableness (*b* = 0.116, *p* < .05) significantly positively affected academic burnout. However, Honesty-Humility, Emotionality, and Conscientiousness were not associated with academic burnout.

### Comparison between academic engagement model and burnout model

When comparing the academic engagement and burnout models in Table 3, we observed significant differences among the three pathways. Specifically, ER_reap predicted academic engagement (*b* = 0.106, *p* = .050) but not academic burnout (*b* = − 0.015, *p* = .552), whereas Agreeableness did not predict academic engagement (*b* = − 0.072, *p* = .546) but was significant for academic burnout (*b* = 0.116, *p* < .05). Similarly, Openness was a significant predictor of academic burnout (*b* = − 0.065, *p* < .05) but not academic engagement (*b* = 0.081, *p* = .053).

**Table 3:** Differential path estimates of academic engagement and burnout models

Exogenous		Endogenous	Estimates	Std Estimates	*p*-value
ER_reap	→	Engagement	0.106	0.165	0.050*
ER_reap	→	Burnout	−0.015	−0.047	0.552
AGREE	→	Engagement	−0.072	−0.058	0.546
AGREE	→	Burnout	0.116	0.191	0.046*
OPENN	→	Engagement	0.081	0.137	0.053
OPENN	→	Burnout	−0.065	−0.147	0.015*

*p < .***05***

Note: ER_reap: cognitive reappraisal of Emotion Regulation, AGREE: Agreeableness, OPENN: Openness to experience

## Discussions

This study specifically explored the relationship between personality traits, academic engagement, and academic burnout, with a primary emphasis on academic engagement and burnout. The results have shown that certain personality traits directly and indirectly influence both academic burnout and academic engagement, with emotion regulation playing a mediating role. Particularly, it was revealed that among the personality traits examined, Extraversion had a significant impact on both burnout and engagement among dental students. In the academic engagement model, only the personality trait Extraversion had a direct effect on engagement and had an indirect effect through mediating ER_reap. On the other hand, in the academic burnout model, not only Extraversion but also Agreeableness and Openness were found to have a direct effect on burnout. These results suggest that academic burnout is influenced by a broader range of personality traits compared to academic engagement.

Previous studies have frequently highlighted the close correlation between specific personality traits and academic achievement in healthcare profession education. Doherty and Nugent [45] discovered that social factors such as Extraversion are associated with advanced cognitive abilities in medical education. In the online environment, even in the context of COVID-19, Extraversion was found to positively predict students’ learning engagement and performance [46]. This is consistent with the established correlation between learning performance and academic engagement. The findings of this study are also consistent with those of previous research [47], which suggests that students who score higher on the trait of Extraversion in online environments are at a lower risk of burnout. Extroverted students may reduce the likelihood of academic burnout by demonstrating a greater willingness to seek assistance from others when encountering learning difficulties.

Students with high Agreeableness were found to have a significantly positive predictive value for academic burnout. This personality type values social relationships and tends to be influenced by others [47], with some stress associated with academic work alleviated through positive social interactions [48]. As such, it is plausible that students with high Agreeableness would be more susceptible to academic burnout when their social activities are disrupted by the COVID-19 pandemic. The fact that Agreeableness predicted academic burnout but not academic engagement suggests that while students scoring higher in Agreeableness may be more vulnerable to burnout, this does not necessarily translate into increased academic engagement.

Those who exhibit high levels of Conscientiousness are more likely to be organized, to plan ahead, and to think more carefully. Such individuals may not be overburdened with work and tend to adhere to learning tasks [49]. Considering that Conscientiousness appears to be a significant personality trait associated with individual achievement [45], and given that this study was conducted in the context of COVID-19 pandemic, it can be inferred that our results of this study are due to a reduction in academic engagement and increased fatigue in the online class setting compared to the traditional class setting, and vice versa for some students. How students perceive the online class setting can affect their academic engagement and academic burnout.

The literature suggests that Openness to experience generally has a positive effect on academic performance, though findings vary [50]. Yu [48] observed that individuals with higher Openness to experience tend to adapt better to technology used in online learning. Our study similarly found that Openness to experience is negatively associated with burnout, implying that students with higher Openness are less likely to experience academic burnout. However, we found no significant association between Openness and academic engagement, which may reflect the timing of our study in 2021 when online classes were no longer new. These results align with Audet’s findings [51], where students with higher Openness initially showed increased engagement during the Fall 2020 semester, when online learning was novel. However, this advantage dissipated by the Winter 2021 semester, as online classes had become routine.

Additionally, emotion regulation had only a modest effect on academic Engagement and Burnout. It was observed that ER_reap had a significant effect on academic engagement, but ER_supp did not have a significant effect on academic engagement and burnout. This may be explained by the complexities associated with emotion regulation and its measurement, as emotion regulation is a multi-dimensional concept that encompasses both cognitive and non-cognitive facets [25]. Research indicates that reappraisal is generally more effective and adaptive than suppression, as suppression can allow unresolved negative emotions to accumulate, demanding ongoing cognitive and emotional resources to manage [52]. Our findings support this, suggesting that suppression is an ineffective strategy for promoting academic engagement, especially in online settings where face-to-face pressures are reduced.

Doulougeri et al. [53] found that students did not utilize any strategies to regulate negative emotions, such as shock and surprise. Specifically, they found that Suppression of emotion regulation in this way was associated with more intrusive or ruminative thoughts about the event, as well as guilt. Therefore, it seems reasonable to assume that suppression was not a predictive factor in academic engagement, and by extension, academic burnout.

Although both emotion regulation strategies did not show a significant relationship with academic burnout in this study, it is important to note that regarding the predictive relationship between emotion regulation and clinician burnout, the importance of emotion management skills such as self-regulation and self-management is clearly essential. These skills help health professions more effectively manage occupational stress and the risk of burnout [29]. Emotion regulation is thought to simultaneously protect the individual from recurring stressors or strains, thereby helping them to achieve external psychological well-being [54]. Simon and Durand-Bush [55] argue that emotion regulation strategies have significant implications for maintaining purpose and meaning in life, both generally and occupationally. A cognitive behavioral therapy program approach has also been applied to dentists, demonstrating significant improvements in mental health issues such as stress, burnout, and depression [56].

While academic engagement and burnout are sometimes categorized within the broader concept of ‘academic well-being,’ it is argued that they are multidimensional and distinct, rather than opposite ends of a spectrum [10]. This distinction is crucial, as individuals can experience elements of both engagement and burnout simultaneously [57]. For instance, the Maslach Burnout Inventory (MBI) might indicate both negative and positive states within the same timeframe [10]. Academic achievement correlates with engagement levels but not necessarily with burnout, and vice versa, highlighting that these constructs may be influenced by personality traits differently. This point is also supported by our findings of differential structural paths between the research models of academic engagement and burnout. Thus, treating engagement and burnout as distinct dimensions allows for a better understanding of their effects and the development of strategies to enhance engagement while mitigating burnout.

Given that burnout often persists into the workforce from its origins in student life, it is crucial to cultivate emotional regulation practices during student years [1, 3]. A systematic review on emotion regulation and burnout in doctors found that self-regulation techniques, including mindfulness, were associated with reduced burnout [25]. Mindfulness, specifically, involves focusing on present-moment awareness, which helps in managing stress and reducing burnout [58]. While no substantial short-term changes were observed, post-training stress and decreased burnout tended to correlate following mindfulness training [58, 59]. Consequently, it is imperative that educational institutions implement programs designed to facilitate the development of emotion regulation skills.

The limitations of this study must be acknowledged, and caution is needed in interpreting the results. Firstly, it is important to note that this survey was conducted just before the midterm exams in the first half of 2021, during which most classes were conducted online due to the COVID-19 pandemic. These circumstances may have influenced students’ overall levels of academic engagement and burnout. Secondly, since this study recruited participants from a single university, it was challenging to account for the influence of various factors that can affect academic achievement, such as economic status, urban conditions, and academic infrastructure. Future research should include participants from more diverse backgrounds to enhance the generalizability of the results. Finally, our study is cross-sectional design. Future longitudinal studies are also necessary to confirm the existence of a strong causal relationship between variables. Furthermore, it would be meaningful to analyze the results by grade level, rather than conducting an overall student analysis to provide a more detailed examination of the results.

## Conclusions

This study has identified specific personality traits that exert both direct and indirect influences on academic burnout and engagement, with emotion regulation serving as a key mediating factor. Extraversion was found to have a significantly negative association with burnout levels and a significantly positive association with engagement levels among dental students. In the engagement model, Extraversion was the only personality trait to exert a direct positive effect on engagement and also influenced engagement indirectly through cognitive reappraisal. In contrast, in the burnout model, Extraversion and Openness to experience both directly contributed to higher burnout levels. Additionally, Agreeableness was shown to have a direct positive association with burnout levels. This underscores the nuanced role that personality traits play in academic engagement and burnout, highlighting the importance of considering individual differences in educational strategies to foster engagement and mitigate burnout.

## Data Availability

The datasets of this article are available from the corresponding author on reasonable request.

## Abbreviations

HOHUM: Honesty-Humility
EMOTI: Emotionality
EXTRA: Extraversion
AGREE: Agreeableness
CONSC: Conscientiousness
OPENN: Openness to experience
ER_reap: Cognitive reappraisal of Emotion Regulation
ER_supp: Suppression of Emotion Regulation
